# Effects of ionizing radiation on the immune system with special emphasis on the interaction of dendritic and T cells

**DOI:** 10.3389/fonc.2012.00102

**Published:** 2012-08-24

**Authors:** Katrin Manda, Annegret Glasow, Daniel Paape, Guido Hildebrandt

**Affiliations:** ^1^Department of Radiotherapy and Radiation Oncology, University of RostockRostock, Germany; ^2^Department of Radiotherapy and Radiation Oncology, University of LeipzigLeipzig, Germany

**Keywords:** dendritic cells, T cells, ionizing radiation, low dose, immune system

## Abstract

Dendritic cells (DCs), as professional antigen-presenting cells, are members of the innate immune system and function as key players during the induction phase of adaptive immune responses. Uptake, processing, and presentation of antigens direct the outcome toward either tolerance or immunity. The cells of the immune system are among the most highly radiosensitive cells in the body. For high doses of ionizing radiation (HD-IR) both immune-suppressive effects after whole body irradiation and possible immune activation during tumor therapy were observed. On the other hand, the effects of low doses of ionizing radiation (LD-IR) on the immune system are controversial and seem to show high variability among different individuals and species. There are reports revealing that protracted LD-IR can result in radioresistance. But immune-suppressive effects of chronic LD-IR are also reported, including the killing or sensitizing of certain cell types. This article shall review the current knowledge of radiation-induced effects on the immune system, paying special attention to the interaction of DCs and T cells.

## INTRODUCTION

The interactions of dendritic cells (DCs) and T lymphocytes are a link between the innate and adaptive cell-mediated immunity. Therefore, radiation-induced disturbances may have serious consequences on the whole immune system. This article provides an overview of DC and T cell function and particularly reviews the effects of low-dose ionizing radiation (LD-IR; <1 Gy) and high-dose ionizing radiation (HD-IR; ≥1 Gy) exposure on the interrelationship of both cell types. Controversial data on the immune-modulatory effects of LD-IR and current knowledge about the immune-suppressive and pro-inflammatory effects of HD-IR are discussed in detail, together with the putative mechanisms behind them. Clinically relevant immunological aspects of ionizing radiation (IR) are presented and the possibility of their exploitation in combined immunotherapy are elucidated.

## DENDRITIC CELLS

Dendritic cells are antigen-presenting cells (APCs) which play a crucial role not only in inducing adaptive immune response to foreign antigens (Ags), but also in maintaining T cell tolerance to self-Ags, thus minimizing autoimmune reactions (Banchereau and Steinman, 1998). All DCs are derived from hematopoietic stem and progenitor cells in the bone marrow and give rise to distinct progenitors, which can be found in the blood, lymph, thymus, and most visceral organs. Their further development comprises differentiation into DC subsets, activation and maturation finally resulting in Ag presentation (Alvarez et al., 2008). Newly differentiated DCs are responsible for efficient Ag capture via a variety of mechanisms including macropinocytosis, endocytosis (Lim and Gleeson, 2011), or phagocytosis (Matsuno et al., 1996); these DCs are considered to be immature. To initiate immunity, immature DCs migrate throughout the body in order to take up several Ags, but expression of major histocompatibility complex (MHC) gene products and co-stimulatory molecules such as cluster of differentiation (CD) 80 and CD86, and thus presentation to T cells, is initially weak (Mellman and Steinman, 2001). Upon arrival at secondary lymphoid organs, such as draining lymph nodes (dLN) and the spleen, they have to undergo a maturation process initiated by several environmental stimuli or danger signals including bacterial DNA (Sparwasser et al., 1998) or viral products and proinflammatory cytokines (Mellman and Steinman, 2001). Maturation is characterized by an increase in surface marker expression responsible for co-stimulation, including CD40, CD54, CD58, CD80, CD83, and CD86 (Banchereau and Steinman, 1998; Faries et al., 2001; Würtzen et al., 2001) along with the ability to present Ag more effectively to T cells. As a consequence of maturation, Ag uptake of DCs is reduced through a loss of Ag receptors and down-regulation of phagocytosis (Albert et al., 1998).

## T CELLS

T lymphocytes are main player in the cell-mediated adaptive immune response. After migration of progenitor T cells from the bone marrow to the thymus T cells differentiate, resulting in the expression of the typical co-receptors CD4, CD8 and the assembly of functional T cell Ag receptors (TCRs). T cells then undergo a positive and negative selection process based on MHC receptor restriction and on the affinity threshold of their TCR to self-peptides presented by MHC molecules on the thymic epithelial cortical cells (Starr et al., 2003; Arens and Schoenberger, 2010). Naive, but mature T cells migrate to the secondary lymphoid organs where they survey the Ags presented by APCs. The TCRs recognize Ag fragments bound to MHC molecules on the surface of an APC. As a consequence of Ag binding and interaction with cytokines and co-stimulatory molecules, naive CD4^+^ or CD8^+^ T cells become activated, proliferate, and differentiate into effector T cells (Lee et al., 2012).

Whereas the majority of CD4^+^ T cells are helper T (T_h_) cells selectively binding to MHC class II proteins, the majority of CD8^+^ T cells are cytotoxic T cells (CTLs) restricted to binding to MHC class I proteins (Banchereau and Steinman, 1998). T_h_ cells assist other cells of the immune system such as B cells and macrophages and can be further categorized into T_h_1, T_h_2, and T_h_17 subsets (Reiner, 2007). T_h_1 cells are primarily involved in cell-mediated inflammatory reactions including activation of macrophages and CTLs. T_h_2 cells aid the humoral and allergic arms of the immune response and are associated with eosinophilia (Kimber and Selgrade, 1998). T_h_17 cells are important for attacking extracellular microorganisms by activating neutrophils with interleukin (IL)-17. In addition to T_h_ cells, CD4^+^ T cell also comprise subsets which have the ability to regulate inflammatory immune responses and are therefore termed regulatory T cells (T_reg__s_; Lee et al., 2012). These cells express CD25, the IL-2 receptor and play a major role in maintaining immunological self-tolerance (Sakaguchi, 2004). Subsets of T_reg_ have also been demonstrated to inhibit both T_h_1 and T_h_2 functions, crucial to the outcome of infections and inflammatory diseases (Xu et al., 2003).

The main function of Ag-specific CD8^+^ T cells (CTLs) is to eradicate infected or tumor cells through the release of cytolytic molecules and CD95 ligation, eventually leading to the programed cell death (apoptosis) of the target cell. Besides antigenic stimulation (signal 1) and co-stimulation by APCs (signal 2), inflammatory cytokines such as IL-12 and type I interferons (IFNs) are important for driving effector T cell expansion and function (Arens and Schoenberger, 2010). CD4^+^ T cells effectively support CTL response, especially during the secondary expansion phase, and by generating long-term CTL immunity (Behrens et al., 2004).

As part of the adaptive immune response, both CD4^+^ and CD8^+^ T cells comprise also memory T cell subsets which have already encountered Ag during a prior infection and escaped apoptosis. After a second encounter with Ag or pathogen, memory T cells are able to work quickly without even requiring proliferation (Bevan, 2011). They are further categorized into effector memory T cells (T_EM_) and central memory T cells (T_CM_) based on their capacity to migrate to secondary lymphoid tissue (T_CM_) and infected or inflamed peripheral sites (T_EM_). The main distinctive feature of the two memory T cell subsets is the expression of chemokine receptor 7 (CCR7), which exists on T_CM_ but is lacking on T_EM_ cells (Sallusto et al., 1999).

## INTERACTION OF DCs AND T CELLS

The presentation of Ags by DCs plays a crucial role in effective T cell activation and initiation of an adaptive immune response. Naive CD8^+^ cytotoxic and CD4^+^ T_h_ cells circulate through secondary lymphoid tissues where they meet activated mature DCs presenting processed Ags to them via MHC class I and II molecules, respectively. Both cell types need to interact physically to induce T cell activation and proliferation. The subsequent outcome of T cell activation depends on the activation state of DCs. Activated, mature DCs induce T cell priming, whereas resting, non-activated but fully differentiated mature Ag-presenting DCs may induce tolerance (Tan and O’Neill, 2005; Hugues, 2010). The latter is a process which is required to eliminate self-reactive T cells in the thymus during a process known as central tolerance. However, some self-reactive T cells often bearing low affinity TCR for self-Ags escape clonal deletion in the thymus. A number of tolerance mechanisms have evolved in the periphery to prevent autoimmune disease. DCs capturing and presenting numerous self-Ags to T cells in secondary lymphoid tissues are an important part of this peripheral tolerance (Walker and Abbas, 2002).

The current model of T cell activation in general requires three signals. The first signal is the establishment of a cellular contact between a T cell and a DC occurring through TCR interactions with MHC complexes present on the DC surface. In this process, CD4^+^ T_h_ cells can effectively support the Ag-specific CD8^+^ CTL responses via activation of CD40 on DCs when both T_h_ cells and CTLs recognize Ag on the same DCs. The second signal comprises the engagement of different receptor–ligand bindings such as those of co-stimulatory and intercellular adhesion molecules (ICAMs). Important co-stimulatory molecules are CD80 and CD86, expressed on activated but not on resting APCs, which need to bind to the cell surface receptor CD28 on T cells for effective T cell activation and to cytotoxic T lymphocyte antigen 4 (CTLA-4) for suppression. These interactions finally lead to the third signal consisting of the secretion of mediators. The integration of all signals finally matches the outcome of T cell activation, resulting in the clonal expansion and differentiation of naive T cells into effector and memory T cells (Sharpe and Abbas, 2006; Arens and Schoenberger, 2010; Hugues, 2010).

Given the important role of DC and T cell interaction in the adaptive immune responses, it is not surprising that many pathogenic microorganisms exert immunomodulatory effects that may impair the ability of DCs to initiate T cell responses. Virus-induced interference with Ag presentation pathways, induction of cytopathogenesis, T_h_1/T_h_2 cytokine shifts, and CD4 depletion are examples of this (Clerici and Shearer, 1993; Arens and Schoenberger, 2010).

## RADIATION-INDUCED EFFECTS ON THE IMMUNE SYSTEM AND THE INTERACTION OF DCs AND T CELLS

Since the spleen is a very highly radiosensitive organ (Gridley et al., 2009), cells of the immune system are considered to be among the most highly radiosensitive cells. The biological effects of IR are not completely understood, especially the effect of LD-IR. For a long-time IR was assumed to act mostly on target cells. DCs are one of the immune cells which have been studied the most. Nearly all processes mediated by DCs depend on their differentiation and maturation state. These processes involve migration to peripheral lymphoid organs as well as expression of MHC molecules, co-stimulatory molecules and cytokines resulting in T cell stimulation. Thus, IR-induced changes in the state of DC maturation and activation would affect the whole immune system. Additionally, the radiosensitivity of T cells generally depends on their state of activation. Resting (non-activated) lymphocytes are much more affected by IR than their activated counterparts (Anderson and Warner, 1976). Apart from these targeted effects, in recent decades the indirect (non-targeted) effects of IR such as bystander effects, adaptive response, abscopal effect, and genomic instability, have also been described. The reported non-targeted cellular responses to IR were modulating inflammatory and immune responses (Hildebrandt, 2010). The response of the immune system to IR depends, however, on the dose and the dose rate (Amundson, 2008) as well as on the irradiation quality and the immune cell types (Rödel et al., 2012).

### EFFECTS OF HIGH-DOSE IRRADIATION

For this review HD-IR was defined as using single doses of 1 Gy or more. The immunosuppressive effects of HD-IR on the immune system are well known. Epidemiological and patient data show that acute radiation syndrome occurs after whole-body irradiation (WBI) of more than 1 Gy delivered at a high-dose rate (Goans and Waselenko, 2005). Higher radiation doses (>2 Gy) result in a massive killing of blood cells such as lymphocytes (Donnelly et al., 2010) and even in a halting of the proliferation of hematopoietic progenitors, thereby causing hematological crisis (Goans and Waselenko, 2005). Dainiak et al. (2003) stated that the shortage of leukocytes finally leads to suppression of immune function, increasing the risk of infections and impairing wound healing following irradiation with doses more than 2–3 Gy. Besides immunosuppression, one of the most common effects of HD-IR is the induction of pro-inflammatory processes. Long-term studies conducted on blood samples taken from survivors of the atomic bombings of Hiroshima and collected between 1995 and 1997 showed altered tumor necrosis factor alpha (TNF-α), and INF-γ levels which increased with rising doses (Hayashi et al., 2005). Nevertheless, also anti-inflammatory cytokine levels, such as that of IL-10, were increased with increasing dose.

Pecaut et al. (2001) published animal data which correlates with the situation in humans; they showed that HD-IR led to a loss of spleen and thymus mass. They observed decreasing leukocyte and lymphocyte (CD4^+^ as well as CD8^+^ subpopulations) numbers in the blood and spleen of mice treated with WBI, applying doses up to 3 Gy.

*In vitro* investigations showed radiation-induced (20 Gy, ^137^Cs source) alterations of human DC function, including a less efficient Ag-presenting function (Anton et al., 1998) and a lower capacity of induction of T cell proliferation (Cao et al., 2004). There is evidence that very HD-IR (single dose of 30 Gy) reduces the co-stimulatory receptor expression in immature DCs (Reuben et al., 2004) and down-regulates the expression of CD86 and CD80 on human DCs compromising their ability to capture and present Ag (Cao et al., 2004). These results were supported by Liao et al. (2004), who found, in murine DCs treated with 10 Gy, a down-regulation of proteasome activity which is responsible for the processing of Ags for presentation. Also, alterations in the cytokine release of T cells were found in a co-culture with irradiated human DCs compared to naive (unirradiated) DCs (Cao et al., 2004). These alterations include increased IL-2 and IL-4 levels resulting in a lower capacity of HD-IR treated DCs to promote T cell proliferation efficiently. Liao et al. (2004) found marginally decreased MHC class II and CD86 expression on murine DCs 24 h after HD-IR with 2 or 10 Gy. There are also studies revealing a shift of T_h_ cells toward T_h_2 instead of T_h_1 differentiation after HD-IR, paralleled by changes in the cytokine expression profile (Han et al., 2002; Park et al., 2005). It has been suggested that gamma irradiation regulates the level of cytokine-mediators through transcriptional modulation, including signal transducer and activator of transcription (STAT) phosphorylation (Han et al., 2002, 2006). Members of the STAT proteins are involved in the activation of different cytokines and mice with altered STAT genes were shown to have enhanced T_h_2 response and consequently, a lack of T_h_1-type cytokines. This shift toward T_h_2 differentiation after HD-IR may be important – Westermann et al. (1999) suggest that T_h_2 cells might play a critical role in the pathogenesis of radiation-induced pneumonitis in rats. Furthermore, various organ-specific autoimmune diseases were reported after fractionated total lymphoid HD-IR (2.5 Gy, 17 times) on mice, probably caused by modification of T cell dependent control of self-reactive T cells (Sakaguchi et al., 1994).

#### Clinical aspects of high-dose radiation

High-dose ionizing radiation is applied in approximately 50% of all cancer patients and represents a major component of standard cancer therapy (Baskar et al., 2012). Recent investigations have demonstrated that the success in cancer treatment is contingent upon synergy of radiotherapy with the host’s immune response. Whereas radioimmunotherapy uses antibodies directed against specific tumor Ags labeled with radioisotopes to deliver the radiation directly to the tumor, new combination approaches may use the effects of local HD-IR alone or especially in combination with further immune stimulation on the tumor cells or vasculature for a more efficient immune response.

High-dose ionizing radiation has been shown to up-regulate stress proteins which can function as neoantigens in target cells. These then might attract APCs or NK cells which have the capacity to recognize stress ligands and to selectively clear damaged or stressed cells by phagocytosis or cytolytic activity (Hallahan et al., 2001; Gastpar et al., 2005; Formenti, 2010). Also, radiation-induced distinct forms of cell death have been shown to be highly immunogenic and has already been suggested to improve the poor inherent capacity of glioma cells to stimulate APC response in DC vaccination approaches (Ehtesham et al., 2004). It is thought that the exposure of pro-apoptotic proteins like calreticulin triggers the effective recognition and phagocytosis of tumor cells by DCs, leading to CTL response. In the brain, an immunologically privileged area, HD-IR treatment of brain tumors contributes toward the disruption of the blood–brain barrier (Nordal and Wong, 2005) and might synergize with vaccination therapy by facilitating the entry of immune cells. Radiation-induced “danger,” death and inflammatory signals as increased MHC class I, Fas/CD95 expression and chemokine release can additionally attract activated T cells (Demaria et al., 2005a; Formenti, 2010).

Clinical results show that standard radiotherapy alone is inadequate in converting the existing immune suppression/tolerance of an established tumor. So far combination of radiotherapy with immunotherapy remains understudied in the clinic, but promising response rates have been achieved in preclinical settings including melanoma, mammary, and colon carcinoma. First clinical trials are underway (Formenti, 2010) and surely more will follow as soon as the clinical application of immunotherapy for cancer (Scott et al., 2004; Omay and Vogelbaum, 2009) moves forward.

In a murine model irradiation of cutaneous melanomas prior to resection led to a reduction in lung metastasis after systemic challenge with untreated melanoma cells (Ma et al., 2011). Similarly, immune-mediated inhibition of lung metastases after treatment with local radiation was described in a murine metastatic mammary carcinoma model using CTLA-4 blockade (Demaria et al., 2005b). Therefore we may assume that the host’s immune response against the irradiated tumor might be the central player of the abscopal (outside the target) effects of radiotherapy if negative regulators of immune response are inhibited and the tumor-specific effector T cells target cancer cells at metastatic sites (Formenti, 2010).

### EFFECTS OF LOW-DOSE IRRADIATION/CHRONIC LOW-DOSE IRRADIATION

The risk of cancer development or other effects of IR with low doses (<1 Gy; LD-IR) is often extrapolated from the results of epidemiological studies on more highly exposed individuals using the linear, no-threshold (LNT) hypothesis. The LNT model assumes that the radiation-induced risk of cancer is proportional to dose, with no threshold (Puskin, 2009). However, there are many studies indicating that dose–response curves for LD-IR are non-linear, displaying discontinuous dose dependencies, and that they reflect the hypersensitivity of cells to LD-IR not being predictable by extrapolation of the HD-IR response (Kern et al., 1999; Zaichkina et al., 2004; Rödel et al., 2007).

The underlying mechanisms of this discontinuous dose response remain unclear and may result from various overlapping individual processes (Rödel et al., 2010). One possible explanation may be that DNA structures might not be affected as harmfully by LD-IR, thus facilitating a better repair capacity (Rödel et al. 2002). But also epigenetic mechanisms like DNA methylation (Ma et al., 2010) or a differential protein expression (Rödel et al. 2012) may be possible explanations.

Since there is no general definition of LD-IR, we categorized the following paragraph into chronic IR with low-dose single fractions resulting in high total doses (>1 Gy; see Chronic Low-dose Irradiation with Total Doses of More Than 1 Gy) and chronic IR as well as single fraction IR with low total doses (≤1 Gy; see Single Low-dose Irradiation and Chronic Low-dose Irradiation with Total Doses of 1 Gy or Less).

#### Chronic low-dose irradiation with total doses of more than 1 Gy

In contrast to HD-IR, reports on the effects of LD-IR on the immune system are controversial. There are various animal studies showing that chronic low-dose irradiation with total doses of more than 1 Gy may lead to immunosuppression. Underlying mechanisms were revealed by Yagunov et al. (1998) and comprise a deficiency of hematopoietic stem cells, accelerated cell cycling of bone marrow precursors, or a decreased cell viability of mature blood cells in rats leading to ineffective hemopoiesis. These data were confirmed by studies of Seed et al. (2002) who found a suppression of blood leukocyte levels in dogs. Investigations of the blood samples of 50 radiology workers (age 21–57 years) exposed to long-term LD-IR showed decreased immunological parameters including lower levels of CD4^+^ T lymphocytes as well as decreased total immunoglobulins (IgA, IgG, IgM) compared with non-exposed volunteers (Godekmerdan et al., 2004).

Other reports reveal immune stimulatory effects of chronic LD-IR in animals, including stimulation of growth rates in mice or rats (summarized in Luckey, 1982) and prolongation of the life span in MRL-*lpr/lpr* mice (Ina and Sakai, 2004). Ina and Sakai (2005) found increased numbers of CD4^+^ cells as well as CD8 molecules on the surfaces of CD8^+^ T cells after beginning with continuous WBI of C57BL/6 mice with low doses (1.2 mGy/h). The authors suggest that chronic LD-IR may be able to induce a moderate, but not excessive activation of the immune system.

#### Single low-dose irradiation and chronic low-dose irradiation with total doses of 1 Gy or less

Reports on single-fraction LD-IR or chronic LD-IR with total doses of 1 Gy or less are also contradictory. Recently Jahns et al. (2011) showed that LD-IR (0.5 and 1 Gy) of human DCs and T cells in co-culture lead to a decrease of T cell proliferation, which may suggest a suppressing effect on the immune system. In contrast, no changes in T cell proliferation were induced by IR of DCs alone. They also found no significant changes in DC cytokine release and reported similar to Shigematsu et al. (2007) no modulation of activation marker or co-stimulatory molecule expression, such as CD1a, CD40, CD80, CD86, ICAM, or MHC class II in murine DCs alone, treated with several irradiation doses (0.02–1 Gy). Hence, the authors suggested that LD-IR has no effect on the maturation of DCs.

*In vivo* studies demonstrated an increased tumor latency of lymphomas in radiation-sensitive, cancer-prone heterozygous *TRP53* mice (Mitchel et al., 2003) and a reduction of leukocyte adhesion (which was maximal at a dose of 0.3 Gy) in C75BL/6 mice (Arenas et al., 2006) were reported. Furthermore, suppression of metastasis could be confirmed in tumor-bearing rats after 0.2 Gy WBI; this was attended by an increased expression of genes coding for TNF-α and IFN-γ and a decreased expression of transforming growth factor beta (TGF-β; Hashimoto et al., 1999). The authors suggested immune augmentation as a reason for the antitumor effect of LD-IR. Bogdándi et al. (2010) could demonstrate *in vivo* that low-dose radiotherapy (LD-RT) has an impact on the functional as well as quantitative parameters of murine splenocytes. They found a moderate decrease in the apoptosis of murine DCs after WBI with low doses of 0.01–0.1 Gy. These observations were likewise associated with alterations of the cytokine milieu, including partial down-regulation of IL-4 and IFN-γ. Molecular changes induced by LD-IR show a distinctly different pattern from those caused by HD-IR (Liu, 2003). Liu et al. (2001) showed stimulated expression of CD80 and CD86 on murine APCs after WBI with 0.075 Gy, and increased IL-12 secretion 4 h after IR. Additionally, they were able to demonstrate that the expression of CD28 on T cells was up-regulated and that of CTLA-4 was down-regulated in early time points after LD-IR. Considering the work of these authors together, in reference to suppressed production of IL-10 these findings indicate immunoenhancement by LD-IR. Since an increase of surface molecules on macrophages and an increased secretion of IL-12 results at both LD-IR and HD-IR, Liu (2003) suggests that the different immune reactions resulting from LD-IR compared to HD-IR might primarily depend on changes of T lymphocytes. This hypothesis is supported by studies of Jahns et al. (2011) who found a decrease of CD25, a typical marker for activated T cells, after IR of human DCs and T cells in co-culture after 0.5 and 1 Gy, whereas they reported no impact of LD-IR on DCs alone (see also above). The authors assume that this is an effect of LD-IR on T cells rather than on DCs.

The expression of leukocyte adhesion molecules such as L-selectin (Kern et al., 2000) as well as that of chemokines such as CCL20 (Rödel et al. 2008), all playing a fundamental role in leukocyte trafficking and thus are involved in the induction of inflammatory processes, is also reduced by LD-IR *in vitro*. Shin et al. (2010) reported about elevated levels of IL-3, IL-4, leptin, monocyte chemoattractant protein (MCP)-1, MCP-5, macrophage inflammatory protein 1 alpha (MIP-1α), thrombopoietin, and vascular endothelial growth factor (VEGF) along with slight reduction of IL-12p70, IL-13, IL-17, and IFN-γ in murine peripheral blood sera after chronic LD-IR with a total dose of 0.2 Gy (0.7 mGy/h). According to the authors, this pattern of cytokine release maybe facilitates the differentiation of naive T cells into T_h_2, but not into T_h_1 cell type.

Further LD-IR studies reported an increased *in vitro* proliferation response to mitogens such as Concanavalin A in lymphocytes, isolated after WBI of mice with 0.02 or 0.75 Gy (Ibuki and Goto, 1994; Liu et al., 1994a). Liu et al. (1994b) also reported a temporary stimulation of the protein kinase C activity of mouse splenic tissue and lymphocyte subpopulations after WBI with 0.75 Gy X-rays. In general, data indicate that immunoenhancement is restricted to a very narrow range of doses and is dependent on investigated endpoints (Safwat, 2000).

There also is evidence that exposure to LD-IR can result in radio-adaptation (reviewed in Jolly and Meyer, 2009). As a consequence of this process, known as “radiation hormesis,” cells are more resistant to subsequent radiation events (Bhattacharjee and Ito, 2001; Mitchel, 2006).

With the current knowledge no threshold dose can presently be defined for the immune-enhancing effects of irradiation (Safwat, 2000). Variations due to the tested endpoints, animal species or the radiation dose rates applied may additionally complicate those investigations.

#### Clinical aspects of low-dose radiation

The clinical acceptance of LD-RT varies worldwide (Seegenschmiedt et al., 2004). Because of reports from the 1960s and epidemiological data about a possible carcinogenic late risk, especially of leukemia, the application of LD-RT is still a subject of controversial debate and less accepted in many countries (Leer et al., 1998). But in several European countries, LD-RT is practiced for the treatment of a variety of inflammatory and painful joint diseases (Hildebrandt et al., 2003; Seegenschmiedt et al., 2004), such as heel spurs (Heyd et al., 2007), osteoarthritis (Hildebrandt et al., 2000) or tendonitis (Adamietz et al., 2010). Total doses of LD-RT comprise 5–10% of those given to tumor patients, assuming different radiobiological mechanisms triggered by LD-RT compared with high-dose radiotherapy (HD-RT; see remarks above). In animal models it was demonstrated that repeated LD-RT can attenuate the pathology of autoimmune diseases. In collagen-induced arthritis mice, used as a model of rheumatoid arthritis, a suppression of IL6 and IL17 production and up-regulation of T_reg__s_ was demonstrated after repeated irradiation with 0.5 Gy (Nakatsukasa et al., 2010). LD-RT may also have a potential therapeutic effect for the attenuation of the pathology of other autoimmune inflammatory diseases, such as multiple sclerosis (MS). In experimental autoimmune encephalomyelitis mice, an established animal model of human MS, suppression of pro-inflammatory cytokines, reduction of CD8^+^ CTLs, and induction of T_reg__s_ could be observed after repeated irradiation with 0.5 Gy (IR once per week for 4 weeks; Tsukimoto et al., 2008).

However, as long as there is insufficient knowledge about the precise biological effect of LD-IR, the old fears of tumor induction will remain. Currently, it is intended to investigate the mechanisms and biological impact of LD-IR on modulation of inflammatory response in the context of a sub project of the European project DoReMi (FP7-249689). Furthermore, several patterns of care studies as well as clinical investigations of anti-inflammatory and analgesic LD-RT in Germany are being conducted (summarized in Rödel et al. 2012). The results of all these investigations may help to gain reconsideration of LD-RT as an alternative option for the treatment of benign diseases, also in the countries where LD-RT is still less accepted.

## CONCLUSION AND OUTLOOK

**FIGURE 1:**
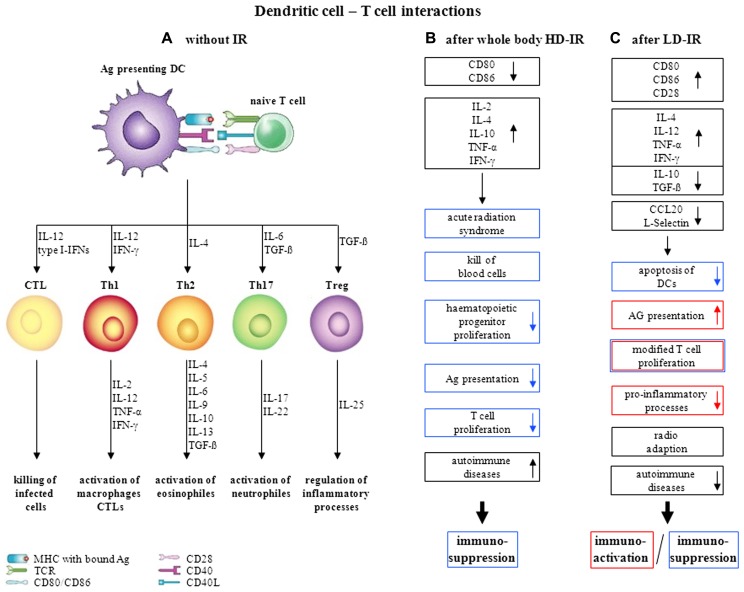
**Dendritic cell–T cell interactions and its variations upon irradiation, (A)** without irradiation (modified after Moser and Leo, 2010), **(B)** influence of whole-body HD-IR, **(C)** influence of LD-IR.

The effects of whole-body HD-IR on the immune system are well characterized, leading in the end to substantial immunosuppression. Underlying molecular mechanisms are inhibition of Ags-presenting function (Anton et al., 1998) by down-regulation of co-stimulatory receptors such as CD80 and CD86 in immature DCs (Reuben et al., 2004), alterations in cytokine release (Han et al., 2006) and radiation-induced depletion or proliferation stop of progenitor cells (Goans and Waselenko, 2005). A consolidated overview of the interactions of DCs with T cells and the effect of whole-body HD-IR on this is given in **Figures 1A,B**. A novel application of IR has emerged in the partnership of localized HD-RT with immunotherapy (Formenti, 2010). Further investigations regarding schedules, fractionation regimens, combination with chemotherapy, and the contribution of the innate immune system are urgently needed to achieve an optimal radiation-induced immunogenicity.

Until now, no consistent position exists with reference to the effects of LD-IR on the immune system. The observed effects are strongly dependent on the range of dose and dose rate as well as on the animal species and even the strain studied. The precise molecular mechanisms underlying single or chronic LD-IR are still a matter of contradictory discussion. As already mentioned above in more detail, on the one hand there are studies indicating immunosuppression, on the other hand studies suggesting stimulation of the immune system. The effect of LD-IR on the interactions of DCs and T cells is summarized in **Figure 1C**. Since LD-RT seems to have little or no effect on immune cells themselves, but rather on the interactions of DCs and T cells, further investigations will have to be made focusing on these findings. Due to several effects interfering with each other, *in vivo* experimental data often show very donor-specific results, necessitating the establishment of reliable *in vitro* models. These should consist of e.g. different immune cell types, ideally in three-dimensional configuration, to reveal the underlying mechanisms more precisely.

## Conflict of Interest Statement

The authors declare that the research was conducted in the absence of any commercial or financial relationships that could be construed as a potential conflict of interest.

## Abbreviations

Ag: antigens
APC: antigen-presenting cell
CCL: chemokine ligand
CCR: chemokine receptor
CD: cluster of differentiation
CTL: cytotoxic T cell
CTLA: cytotoxic T lymphocyte antigen
DC: dendritic cell
dLN: draining lymph node
HD-IR: high-dose ionizing radiation
ICAM: intercellular adhesion molecule
IFN: interferon
IG: immunoglobulin
IL: interleukin
IR: ionizing radiation
LD-IR: low-dose ionizing radiation
LD-RT: low-dose radiotherapy
LNT: linear, no-threshold
MCP: monocyte chemoattractant protein
MHC: major histocompatibility complex
MIP: macrophage inflammatory protein
NK cell: natural killer cell
T_C__M_: central memory T cells
TCR: T cell antigen receptor
T_E__M_: effector memory T cells
TGF: transforming growth factor
T_h_ cell: helper T cell
TNF: tumor necrosis factor
T_reg_: regulatory T cell
STAT: signal transducer and activator of transcription
VEGF: vascular endothelial growth factor
WBI: whole-body irradiation.
